# Third-Generation Behavioural Therapies in the Context of Neurodevelopmental Problems and Intellectual Disabilities: A Randomised Clinical Trial with Parents

**DOI:** 10.3390/ijerph20054406

**Published:** 2023-03-01

**Authors:** David Lobato, Francisco Montesinos, Eduardo Polín, Saray Cáliz

**Affiliations:** 1 Department of Psychology, Universidad Europea de Madrid, 28670 Madrid, Spain; 2 Instituto ACT, 28036 Madrid, Spain

**Keywords:** parenting, psychological flexibility, disability, neurodevelopmental, contextual therapies, Acceptance and Commitment Therapy, parental stress, health

## Abstract

The purpose of this study was to examine how 14 parents of children with autism and intellectual impairments responded to an Acceptance and Commitment Therapy (ACT)-based psychological flexibility intervention programme. A randomised clinical trial was conducted. Parents were randomly assigned to the training programme group (*n* = 8) or waiting list group (*n* = 6). The treatment effect was measured using the 6-PAQ, PSS-14, GHQ-12, and WBSI questionnaires. Changes in interactions were assessed through self-recording, including a baseline to observe the previous functioning. Measures were taken before and after the application of the intervention programme and three months later. After that, the control group was switched to the psychological flexibility programme condition. After the programme’s implementation, we could see a reduction in stress and the tendency to suppress unwanted private events. The impacts also appeared to apply to family interactions, resulting in a rise in positive interactions and a decrease in unfavourable ones. The results led us to think about the importance of psychological flexibility for the parents of children with chronic conditions, facilitating a reduction in the emotional impact derived from parenting and the emission of behaviours that promote the harmonious development of the diagnosed child.

## 1. Introduction

The Diagnostic and Statistical Manual of Mental Disorders 5 [1] proposed a new classification of disorders known as neurodevelopmental disorders (NDDs), a category of conditions that usually emerge in childhood and can be chronic conditions that persist over a lifetime. NDDs includes disorders that manifest in a general way in almost all developmental domains (i.e., intellectual disability or autistic spectrum disorder) and specific domains (i.e., attention-deficit/hyperactivity disorder).

Parenting a child with neurodevelopmental disorders and/or intellectual disabilities can be a stressful and challenging experience, requiring complex skills, which often has repercussions on the family members’ general health. Parents of children with neurodevelopmental disorders and/or intellectual disabilities are more at risk of psychological stress or burnout than other parents [2]. This stress can manifest itself in a lower quality of life [3], health problems [4], emotional disturbances such as depression and anxiety [5], the well-being of the family itself (i.e., physical health, relational and marital satisfaction [6]), or the effectiveness of psychological interventions in the context of neurodevelopmental disorders such as autism and/or intellectual disability [7]. In addition, this parental psychological distress can negatively impact parental practices and family functioning [8] by leading to more hostility and lower parental responsiveness [9]. This psychological distress is further exacerbated if the child presents severe behavioural disturbances [10]. This parental suffering and relational style have a negative impact on the health of children with neurodevelopmental disorders, as they are deprived of adequate exposure to situations that allow for them to develop skills of any kind (social, personal, basic activities of daily living, or other learning). In this way, there is a cumulative effect on the difficulties previously caused by this condition and those derived from the caregiver’s state of health. For this reason, these people reach worse levels of development and global functioning [11]. Therefore, a growing number of studies support the suitability of Acceptance and Commitment Therapy (ACT) for parents who have a child with chronic difficulties such as autism [12], cerebral palsy [13], intellectual disability [14], or chronic pain [15]. The clinical goal of ACT-psychological flexibility (PF), defined as the ability to persist or change one’s behaviour in the service of one’s chosen values while being aware of the situational context and one’s experience at the present moment [16], seems to fit well with the demands of parenting children with chronic conditions [17]. PF has been noted as a modulating variable for the impact of psychological difficulties, parental load, and stress in families of children with neurodevelopmental impairments or disabilities [18,19,20]. In earlier research by our team, it was found that PF was connected to the effects of parental stress and health issues caused by parenting a child with intellectual disability [21]. Likewise, PF intervention programmes for parents reduced stress, improved quality of life, and modified maladaptive interaction repertoires among family members [22,23]. A randomised clinical trial was proposed with the aim of testing whether, after PF training, parents would reduce their stress, health problems, and tendency to suppress unpleasant thoughts and improve their PF. We also wanted to check whether PF training could be extended to interactions with children diagnosed with neurodevelopmental disorders and intellectual disabilities. The aim of the current study was to test the effectiveness of a brief protocol based on psychological flexibility in a group format to improve the psychological well-being of parents raising a child with chronic conditions such as neurodevelopmental disorders.

## 2. Materials and Methods

### 2.1. Design

This study was approved by the European University of Madrid Ethics Committee (CIPI/20/153). The trial was registered on ClinicalTrials.gov (ID: NCT05611554). Randomisation was completed by the Principal Investigator using an online service and randomly sized, permeated blocks to each arm (ratio: 1:1). The trial was a single-centre RCT with two arms, with participants assigned to: (1) psychological flexibility programme for parents (intervention group) or (2) waiting list (control group). Participants were assessed at baseline (pre-treatment), at the end of the programme (post-treatment), and 3 months after the end of the programme (follow-up). Participants on the waiting list condition remained in the study after assessment and received psychological intervention.

### 2.2. Participants

**Figure 1:**
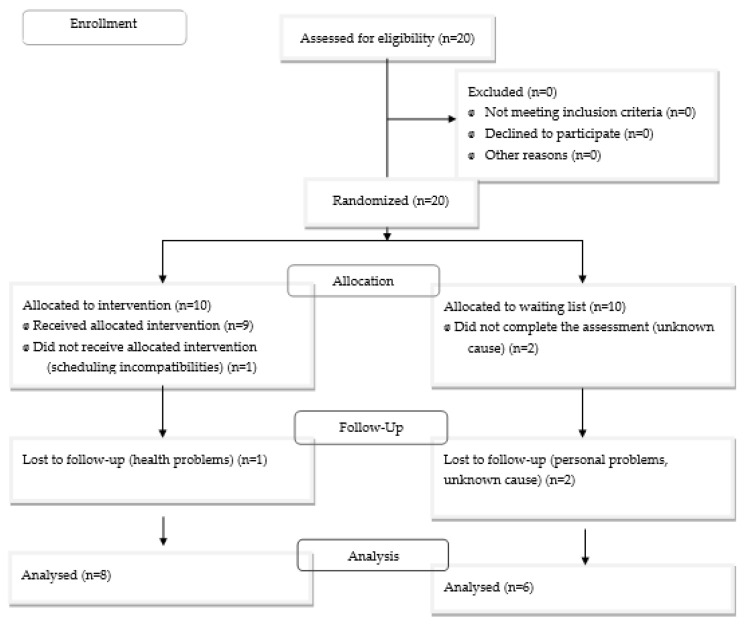
CONSORT flow diagram.

Convenience sampling was carried out based on the availability of participants. The eligibility requirements included being over 18 years old, not currently receiving psychological treatment, being fluent in Spanish, and having a child diagnosed with a neurodevelopmental disorder or intellectual disability. The participants did not receive any financial incentive for participating. Twenty parents were recruited through a non-governmental organization for those affected by neurodevelopmental disorders in the region of Madrid, of whom 10 were assigned to the intervention condition and 10 to the control condition (see CONSORT flow diagram, Figure 1). The majority of the parents were female (80% mothers and 20% fathers), with a mean age of 41.2 years (SD = 7.5). They had an average of 1.4 children (SD = 0.6), with the mean age of 11.9 (SD = 2.1). All children had a diagnosis of autism spectrum disorder, with an intellectual development disorder and mild or no functional language impairment (55%: 30% assigned to intervention condition, 25% assigned to control condition), functional language impairment (30%: 10% intervention condition, 20% control condition), or no functional language (15%: 10% intervention condition, 5% control condition). The sociodemographic details of the study can be obtained from the first author of the manuscript. Two parents in the intervention group withdrew from the study (one due to schedule incompatibility with the programme and one due to health issues), and for the waiting list group—and later intervention after data collection was completed—four parents dropped out or did not finish the study (three for unknown reasons, one due to personal problems).

### 2.3. Measures

The following assessment instruments were used:

*Parental Acceptance Questionnaire (6-PAQ)* [24]. The Spanish version of the 6-PAQ was used to evaluate parental PF [25]. Six processes connected to PF (being present, values, committed action, self as context, cognitive defusion, and acceptance) are assessed in this 16-item questionnaire using a Likert-type scale with four answer alternatives and three flexible response styles (opened, centred, and committed). The scores range from 16 to 64, with higher values indicating higher levels of psychological inflexibility (PI) levels. In studies on psychometric properties of the scale, Cronbach’s alpha scores of 0.81 and a McDonald’s omega of 0.86 were reported [25].

*Perceived Stress Scale (PSS)* [26]. The PSS Spanish adaptation [27] was used to measure the degree of perceived control over life circumstances. In the research on psychometric attributes, Cronbach’s alpha values of 0.727, 0.826, and 0.868 were found. This is a one-dimensional measure with 14 items, and the responses range from 0 (Never) to 5 (Very often) on a Likert-type scale. Higher scores indicate higher stress; the direct values range from 0 to 56 [27].

*General Health Questionnaire (GHQ-12)* [28]. The GHQ-12 is an extensively used tool for the evaluation of psychological well-being. It contains 12 items and the Cronbach’s alpha score for its consistency is 0.85 [28]. Lower psychological well-being levels are indicated by higher scores. It has been validated in Spanish, reporting a Cronbach’s alpha of 0.76 [29].

*White Bear Suppression Inventory (WBSI)* [30]. The tendency to suppress unwanted thoughts was measured using the Spanish validation of WBSI [31]. This is a 15-item Likert scale with five possible responses on a scale from 1 (Completely Disagree) to 5 (Completely Agree), and scores range from 15 to 75. Higher scores show a stronger thought suppression tendency. In the general scale, Cronbach’s alpha values of 0.89 were found, and values of 0.87 and 0.80 were found for its subscales in terms of the scale’s internal consistency [31].

*Behavioural self-monitoring.* Parents documented an estimation of the daily occurrence of two types of behaviours: the punitive–hostile actions of family members towards children with impairments (e.g., yelling, punishing, threatening, insulting, or bullying) and supportive–companion behaviours (e.g., helping, sharing leisure time, and complementing). The occurrence of each type of behaviour was estimated using a single-item Likert-type scale, with 0 indicating never, 1 almost never, 2 sometimes, 3 often, and 4 always. The participants completed 28 daily self-monitoring measures, starting one week before the intervention to create the baseline scenario (BL) and continuing until one week after the intervention. Weekly summations for each participant (one for each type of behaviour) were calculated, and the correspondent group means were used for the statistical analysis.

### 2.4. Procedure

The brief group intervention protocol for family members (9 h) followed a similar structure to the programme implemented in the previous study [23], and focused on (a) values clarification, (b) defusion strategies, (c) mindfulness training, (d) committed action, and (e) psychological acceptance. The theoretical and clinical skills guide by Harris, Páez, Montesinos, and Valdivia [32,33,34] was used as a basis for the therapeutic techniques. The contents of the intervention are listed in Table 1 (if needed, a detailed version of the protocol may be requested from the first author). The intervention was conducted weekly by a trained therapist in the third-generation therapies, who was experienced in working with families (lead author). It consisted of three sessions, each with a duration of three hours, and was conducted in collaboration with an NGO (*Centroconmigo*, Madrid). The self-reports were administered at three different timepoints, before the intervention (pre-treatment), one week after its conclusion (post-treatment), and three months after its conclusion (follow-up). The participants signed the informed consent form, completed their workbooks at each session, participated voluntarily, and followed the program. The participants in the waiting list group (control) completed the same evaluation but did not receive the intervention program until the end of the research.

### 2.5. Statistical Analysis

Pre-treatment, post-treatment, and follow-up (3 months) variables were analysed and described. To evaluate the effectiveness of the intervention protocol, two complementary strategies were used. On the one hand, the scores of the participants on each instrument before the intervention programme were compared to those obtained after the intervention programme (i.e., pre–post) and three months later (i.e., pre–follow-up). For this purpose, a Wilcoxon W test was applied to each comparison. Likewise, the effect size [36] was estimated using Rank–Biserial Correlation. Using the method suggested by Jacobson and Truax, the clinical significance of the changes achieved as a result of the application of the intervention was contrasted [36], which makes it possible to observe whether the change in the variables collected was clinically significant and reliable. Jacobson and Truax consider that a clinically significant change occurs when there is a return to normal functioning and the change becomes part of the functional population [37]. Considering the average population, the authors propose three different criteria, each with their own respective cut-off points, where the first criterion is the one that best suits the present study. This criterion states that the post-intervention scores should be two standard deviations (SD) outside the dysfunctional range. In addition, to verify that the observed change is reliable, they proposed what the authors call the Reliable Change Index (RCI), which is established in standard deviations. If the RCI presents absolute values greater than or equal to 1.96, it can be stated that the change is reliable. The Jacobson and Truax method classifies a patient as recovered if the RCI value is greater than 1.96 and the cut-off point is exceeded, improved if the RCI value is greater than 1.96 but the cut-off point is not exceeded, unchanged if the RCI value is not greater than 1.96, and worsened if the score involves a change that exceeds the RCI value of 1.96 but in an inverse, worsening direction [38].

## 3. Results

Descriptive statistics regarding questionnaires administrations as a function of the group are shown in Table 2.

Table 3 shows the results obtained for the intervention group, and Table 4 shows the results obtained for the control group. The two tables display the results of the comparisons between pre–post and pre–follow up performed using Wilcoxon’s W and the magnitude of the effects are presented.

For the intervention group, a statistically significant change in the expected direction was observed for PI and the suppression of private events before and after the protocol application and during the follow-up phase. In addition, perceived stress showed a statistically significant change during the follow-up phase (i.e., at 3 months). In all cases, and regardless of statistical significance, it can be observed that the scales offer medium or large effect sizes based on the published criteria [39]. For the waiting list group, no statistically significant changes were observed in the evaluation at the end of the programme with the intervention group, nor were they observed during follow-up. Table 5 provides the change data for each of the applied questionnaires for the participant scores before and immediately after the application of the intervention protocol (pre–post) and before and three months after this (pre–follow-up). This information relates to the analysis of the Clinical Significance Measures. Table 6 shows the results for the control group. In order to analyse the most powerful effects, both tables show the number of participants who recovered, did not change, or worsened.

The intervention programme designed for PF training produced clinically significant and reliable behavioural changes in the subsequent variables in the intervention group. Regarding the 6-PAQ (psychological flexibility), changes were found in four participants post-treatment and in four participants at follow-up. No changes were found in the PSS (perceived stress) at post-treatment but three participants improved their perceived stress at follow-up. No changes were found at post-treatment in the GHQ (general health) but one participant improved her general health at follow-up. Improvements were found in two participants in the WBSI (suppression of unwanted private events) at post-treatment and in two participants at follow-up. The following changes in the variables were found in the control group. Regarding the 6-PAQ (psychological flexibility), one participant worsened at post, and one participant worsened at follow-up, and there were no significant changes in the rest of the participants. In the PSS (perceived stress), there were no significant changes in participants at post and one participant worsened at follow-up. In the GHQ (general health), there were no significant changes in participants at post and one participant worsened at follow-up. In the WBSI (suppression of unwanted private events), there were no significant changes in participants neither at post nor at follow-up. Based on the obtained results, the observed changes after the application of the PF-based intervention programme suggest a trend towards increased PF, a decrease in perceived stress, and a trend towards the decreasing suppression of unpleasant private events. For all scales, the effect size was medium–large. Additionally, participants continued to improve during the follow-up period, particularly on stress and health indicators. Finally, repeated ANOVA measurements for both the intervention group and the waiting list group were used to examine the change in parent–child interaction response tendency (supportive/companion and punitive/hostile behaviours) across the four timepoints (BL, W1, W2, and W3) when this specific variable was measured. Table 7 shows the means and standard deviations obtained at each timepoint for the two conditions.

**Figure 2:**
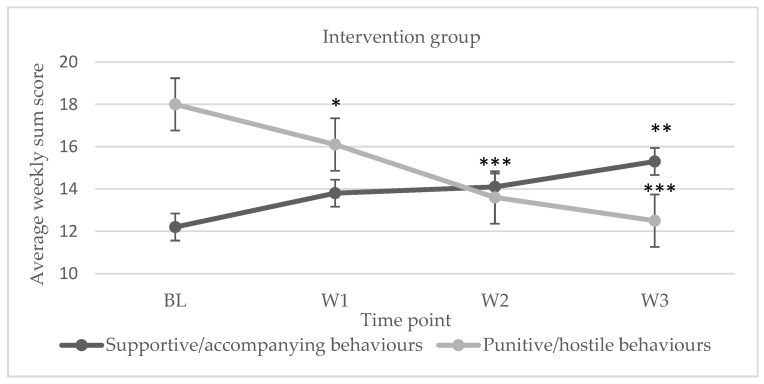
Evolution of family interactions for the intervention group. *Note.* Supportive/accompanying and punitive/hostile interactions over the three weeks the intervention was implemented. Error bars show the standard error of the mean (SEM). * *p* < 0.05, ** *p* < 0.01, *** *p* < 0.001. All the points shown in the graph are statistically significant regarding BL.

**Figure 3:**
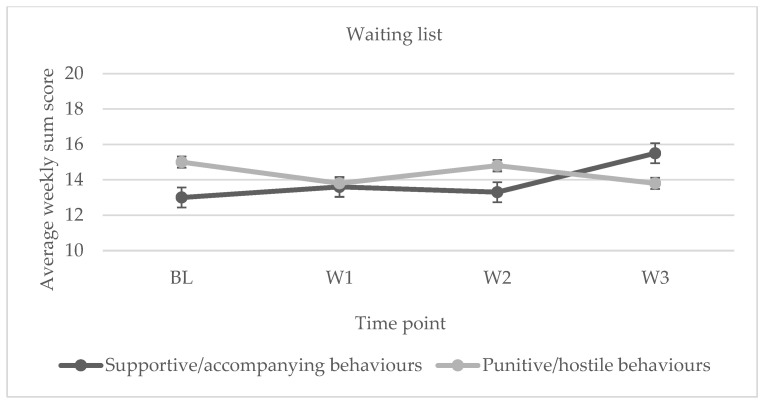
Evolution of family interactions for the waiting list group (control). *Note.* Supportive/accompanying and punitive/hostile interactions over the three weeks the intervention was implemented. Error bars show the standard error of the mean (SEM).

Regarding the results obtained for the intervention condition, in the supportive/accompanying interactions, we observed an increase in responses from the BL to the end of W3, whilst for the punitive/aversive interactions, a decrease was observed from the BL to the end of W3. Figure 2 shows the changes in response trend, with the mean of weekly sum scores of the participants (*n* = 8) being represented on the Y-axis. For the waiting list condition, no change in the response trend was observed except for a slight increase in supportive/accompanying interactions between W2 and W3 (*n* = 6, see Figure 3).

The group receiving the intervention programme showed a statistically significant change in their interaction repertoires for both supportive/accompanying behaviours (F(3.21)= 5.264; *p* = 0.007; η²*_p_* = 0.429; 1 − β = 0.736) and punitive/hostile behaviours (F(3.21)= 27.555; *p* < 0.001; η²*_p_* = 0.797; 1 − β = 0.999). No statistically significant changes were observed for the supportive/accompanying interactions (F(3.21) = 2350; *p* = 0.0114; η²*_p_* = 0.320; 1 − β = 0.324) or punitive/hostile (F(3.21) = 1075; *p* = 0.389; η²*_p_* = 0.177; 1 − β = 0.124) interactions for the waiting list (control) group. Table 8 displays the results.

According to Table 9, the extent of support/accompanying behaviour changes ranges from medium to large in the intervention program group and is statistically significant when comparing the BL and W3 of the intervention. From BL until the end of W3, there is a decline in responses of punitive/hostile behaviours, with the exception of the behavioural change between W2 and W3, where the effect size ranges from medium to large. The most notable changes were between the BL and the last week of the intervention, where a declining trend was seen, similar to the supportive/accompanying interactions. For the waiting list (control) group, no statistically significant improvements were seen in accompanying or punitive/hostile interactions (see Table 10).

## 4. Discussion

The ACT-based intervention for parents of children with neurodevelopmental disorders and intellectual disabilities was followed by a significant reduction in perceived stress, thought suppression, and PI. Additionally, changes in the suppression of private events and PF variables were statistically significant at post-intervention and follow-up. Statistical significance was also reached in changes in perceived stress during the follow-up period. The responses to punitive and hostile interactions with children who have intellectual and neurodevelopmental disabilities considerably decreased while responses to supportive and accompanying interactions rose. The effect size was large–medium for all the studied variables. In addition, the application of the intervention programme in the usual care context of the parents supports the ecological validity of the intervention. The results are similar to those found in recent ACT studies on parents of children with autism and neurodevelopmental disorders [40] and intellectual disabilities of different degrees [14].

The analysis of clinical change, as assessed through the RCI, allowed for us to conduct a more individualised study of behavioural changes. In line with the ACT hypotheses [16], this strengthened the programme’s impact on the variables PF and suppression of unpleasant private events compared to the variables related to stress and general health, which improve during the follow-up phase. This is because the technology derived from contextual–functional science aims to decrease the control of problematic private events and support contact with those unpleasant emotions or thoughts when necessary to ensure public behaviours that contribute to long-term values [41], thus fostering conscious and self-directed parenting. Therefore, the technology does not aim to eliminate, suppress, or control those unpleasant events. The focus on accepting experiences rather than changing them may be particularly beneficial for parents of children with neurodevelopmental impairments and intellectual disabilities, where there is no option to change their children’s condition, and other skills and competencies are required to cope with such situations. Therefore, exercises in ACT are designed to train people to deal with their daily stressors in a functional and flexible way [15], proving more effective in the medium–long term. Although the general health variable shows changes in the expected direction, these are not statistically significant. We hypothesise that this type of effect may be more visible in the long term. Although we obtained similar results (included in the general health variable) in the clinical trial previously conducted by our team, it lacked a control group [23]. This study confirmed the efficacy of the intervention, since the differences between the two groups are very notable, thus providing evidence that the changes are not due to the mere passage of time or the fact that the patients are being evaluated and know that they are participants in a study.

The findings of this randomised clinical trial give us reason to assume that teaching parents in a group setting can be more efficient and effective because certain parents act as role models for others, forming a network of support among family members, particularly among women [42].

Traditional behaviour modification programmes typically offer specific behavioural management instructions for the child with neurodevelopmental impairments and disabilities. However, ACT interventions focus not only on parents’ public behaviour but also on increasing their psychological well-being, not by promoting the control or reduction of discomfort but by incorporating strategies that allow for them to more flexibly relate to their private events, orienting them towards value-based parenting. Therefore, our intervention is focused at the stress derived from raising a child with neurodevelopmental disorders and intellectual disabilities, so that the improvement in the relationship with the unpleasant private events experienced by the parents may also affect the behaviour of the diagnosed child [43]. There is a relationship between parental stress and the behavioural problems of the child [44], so altering this relationship is a priority for the psychologist in interventions with children with developmental disorders. Furthermore, training parents in the more functional management of stressful events (in addition to providing them with tools for the behavioural management of the diagnosed child) has summative effects on outcomes [45]. Therefore, it is essential to incorporate parents and their distress into psychological interventions for children with neurodevelopmental disorders. Along this line, the changes observed in the interactions with the children show an ascending pattern for supportive/accompanying behaviours and a decrease in punitive/hostile behaviours, consistent with the results found in the scientific literature [46]. Following this line of research, by reducing hostility in their interactions, parents respond with more affectionate and caring behaviours, which results in a possible reduction in the behavioural problems of the diagnosed child. Consequently, it seems that PF training orients the parent towards their parental values, delimits the kind of interaction they wish to cultivate with their children, and teaches parents, through various techniques such as defusion and/or acceptance, to overcome the obstacles that emerge when they orient their behaviour towards children with chronic conditions. Therefore, the shift towards a focus on the family system would result in an improvement in the person with neurodevelopmental impairment and disability; this would strengthen the emotional relationships and help the children to reach higher levels of personal development and integration into the community.

However, there are still substantial limitations to this randomised clinical trial. Firstly, there are limitations derived from the small sample size of participants, which were revealed by the power analysis. The vital condition of the family members, the need for care for the diagnosed minors, and the fatigue derived from the journey through the care systems favour low adherence of the participants and the loss of experimental subjects. The results would be more generalisable if they included even more participants. Additionally, the majority of participants were females, in whom, it seems, the training is probably effective. Future research should, however, look at whether the same training is effective in a sample made up primarily of males. The different degrees of disability associated with the diagnosis of autism and the behavioural problems and different levels of punitive/hostile behaviours pre-intervention may be variables that interfere with the results and may affect the analysis of the intervention’s efficacy [47], so future studies could control the diagnosis, the degree of disability, or the external behavioural problems that the child displays. In addition, as is often the case in other studies that evaluate behavioural changes in family training programmes, our trial used a self-report measure (subjective) and did not include prior training in behavioural observation, which could have affected the reliability of the collected data and the results should therefore be interpreted with caution. Finally, one of the main criticisms of behaviour modification programmes is the decrease in long-term effects [48]. To solve this issue, future studies could add standardised questionnaire measures to analyse the possible behavioural changes in the child (e.g., the CBCL: Child Behaviour Checklist) and/or assess whether this change resulting from PF training is maintained over time (e.g., at 6- or 12-months follow-up).

This study aims to continue providing scientific evidence from the perspective of functional contextualism on the relevance of PF and its relationship with improvements in the quality of life of families with children with neurodevelopmental disorders and intellectual disabilities. Thus, PF acts as a core variable to facilitate conscious, self-directed and value-based parenting.

## 5. Conclusions

The findings point to reduced perceived stress, a decrease in psychological inflexibility, and a reduced tendency to suppress thoughts following the intervention, especially at the follow-up. The group intervention protocol’s greatest impacts, meanwhile, are still seen in PF and the suppression of private events.

The findings suggest that ACT family interventions allow for a more flexible response, moving towards what really matters while managing private events that would otherwise get in the way. Although sometimes helpful in the short term, strict behavioural avoidance often involves distancing from other sources of reinforcement, which ultimately leads to a sense of hopelessness and worsens general health problems for parents, who are at greater risk of developing maladaptive coping strategies as they become more stressed. PF training may encourage a reduction in suppressive behaviour and direct the interactions towards parental values. Regarding the changes observed in the present study in the interactions with children diagnosed with neurodevelopmental disorders and intellectual disabilities, there was an increase in supportive/accompanying interactions and a significant decrease in punitive/hostile interactions. Consequently, it seems that training parents in PF reorients the behavioural repertoire towards what is valuable to them, delimiting how they want to act, and shapes, through experiential technology (e.g., mindfulness and psychological acceptance), how to cope with the unpleasant events that arise in that direction. This is operationalised in more pleasant and less aversive interactions, favouring a harmonious climate that allows for meaningful parenting of those with chronic diseases.

## Figures and Tables

**Table 1:** Description of the clinical trial intervention protocol.

Therapeutic Processes							Sessions			
Therapeutic Methods	Acceptance	Defusion	Values	Committed Action	Contact with Present	Self as Context	1	2	3	
Creative hopelessness: assessment of the effectiveness of stress coping through an experiential exercise.	X						X			
“Man in the hole” metaphor [35]										
“The compass metaphor” [32]			X	X				X		
Experiential exercise: What kind of mother or father do I want to be?										
Experiential exercise: What can I do this week to move towards the kind of parent I want to be?										
Observer exercise [34]						X	X	X	X	
Homework: implementing committed actions and self-monitoring.				X			X	X	X	
Exercise: Clarifying values about family/parenting; assessing the importance and value orientation on a scale of 0 to 10.			X					X		
Experiential Exercise: Emotion Mindfulness through physicalisation [32]		X					X			
“Joe the Bum” metaphor [32]										
“Passengers on the Bus” metaphor [35]		X						X	X	
Experiential exercise: Mindful breathing [32]					X			X	X	
Homework: Practicing Mindful breathing exercise (audio recording)							X	X	X	
Experiential exercise: Our values as parents. Sharing the values on which I articulate my actions.			X							
Identifying valuable actions in the presence of internal and external barriers. Experiential exercise: “When...Then”.									X	
Experiential exercise: “The choice point” [32]									X	

**Table 2:** Descriptive statistics of questionnaires scores (pre, post, and follow-up) for both groups.

	Group	Mean	Std. Deviation
PRE 6-PAQ	Control	34.167	10.265
	Intervention	37.625	8.123
POST 6-PAQ	Control	35.500	7.662
	Intervention	31.875	4.853
F/U 6-PAQ	Control	35.833	6.014
	Intervention	31.875	2.900
PRE PSS	Control	33.000	8.695
	Intervention	29.000	6.887
POST PSS	Control	31.833	6.463
	Intervention	27.250	5.574
F/U PSS	Control	33.167	6.882
	Intervention	24.750	4.590
PRE GHQ	Control	20.833	8.305
	Intervention	25.375	3.926
POST GHQ	Control	22.000	7.403
	Intervention	24.250	2.964
F/U GHQ	Control	20.167	3.251
	Intervention	23.125	2.232
PRE WBSI	Control	52.333	11.928
	Intervention	41.625	7.190
POST WBSI	Control	51.000	7.797
	Intervention	37.625	6.022
F/U WBSI	Control	45.500	7.007
	Intervention	36.000	4.309

*Note.* F/U: follow-up.

**Table 3:** Questionnaire scores and comparisons of effect in intervention group.

Measure 1	Measure 2	W	*p*	Power (1 − β)	Rank-Biserial Correlation
PRE 6-PAQ	POST 6-PAQ	36.000	0.014	0.653	1.000
PRE 6-PAQ	F/U 6-PAQ	34.500	0.025	0.580	0.917
PRE PSS	POST PSS	31.000	0.079	0.401	0.722
PRE PSS	F/U PSS	21.000	0.034	0.653	1.000
PRE GHQ	POST GHQ	16.500	0.246	0.273	0.571
PRE GHQ	F/U GHQ	25.000	0.075	0.459	0.786
PRE WBSI	POST WBSI	28.000	0.022	0.653	1.000
PRE WBSI	F/U WBSI	27.000	0.034	0.591	0.929

*Note.* Wilcoxon signed-rank test. F/U: follow-up.

**Table 4:** Questionnaire scores and effect comparisons in control group.

Measure 1	Measure 2	W	*p*	Power (1 − β)	Rank-Biserial Correlation
PRE 6-PAQ	POST 6-PAQ	2.500	0.461	0.162	−0.500
PRE 6-PAQ	F/U 6-PAQ	6.000	0.399	0.132	−0.429
PRE PSS	POST PSS	11.000	0.418	0.148	0.467
PRE PSS	F/U PSS	6.500	0.892	0.058	−0.133
PRE GHQ	POST GHQ	5.500	0.339	0.151	−0.476
PRE GHQ	F/U GHQ	11.500	0.915	0.054	0.095
PRE WBSI	POST WBSI	13.500	0.598	0.086	0.286
PRE WBSI	F/U WBSI	15.000	0.058	0.479	1.000

*Note.* Wilcoxon signed-rank test. F/U: follow-up.

**Table 5:** Total (and percentage) of patients classified into each clinical significance category by the Jacobson–Truax method based on scales’ scores calculated across two time periods in the intervention group.

PRE–POST				PRE–FOLLOW UP		
	Wor	NCS	Rec	Wor	NCS	Rec
6-PAQ	0 (0%)	4 (50%)	4 (50%)	0 (0%)	4 (50%)	4 (50%)
PSS	0 (0%)	8 (100%)	0 (0%)	0 (0%)	5 (62.5%)	3(37.5%)
GHQ	0 (0%)	8 (100%)	0 (0%)	0 (0%)	7 (87.5%)	1 (12.5%)
WBSI	0 (0%)	6 (75%)	2 (25%)	0 (0%)	6 (75%)	2 (25%)

*Note.* Group results obtained as a result of the application of the intervention protocol. Wor: Worsened; NCS: No clinically significant change; Rec: Recovered.

**Table 6:** Total and (percentage) of patients classified into each clinical significance category by the Jacobson–Truax method based on scales scores calculated across two time periods in the control group.

PRE–POST				PRE–FOLLOW UP		
	Wor	NCS	Rec	Wor	NCS	Rec
6-PAQ	1 (16.6%)	5 (83.3%)	0 (0%)	1 (16.6%)	5 (83.3%)	0 (0%)
PSS	0 (0%)	6 (100%)	0 (0%)	1 (16.6%)	5 (83.3%)	0 (0%)
GHQ	0 (0%)	6 (100%)	0 (0%)	1 (16.6%)	5 (83.3%)	0 (0%)
WBSI	0 (0%)	6 (100%)	0 (0%)	0 (0%)	6 (100%)	0 (0%)

*Note.* Group results obtained during the evaluations for participants in the waiting list (control) group. Wor: Worsened; NCS: No clinically significant change; Rec: Recovered.

**Table 7:** Descriptive statistics for estimated supportive/accompanying and punitive/hostile behaviours for both groups.

Intervention Group (*n* = 8)					Control Group (*n* = 6)			
Time	Supportive/Accompanying Behaviours		Punitive/Hostile Behaviours		Supportive/Accompanying Behaviours		Punitive/Hostile Behaviours	
	M	SD	M	SD	M	SD	M	SD
BL	12.25	1.83	18	0.62	13	2.75	15	1.89
W1	13.87	1.35	16.12	1.64	13.66	1.86	13.83	1.83
W2	14,12	1.72	13.62	1.76	13.34	1.36	14.87	1.47
W3	15.37	1.50	12.40	2	15.5	1.37	13.72	1.60

*Note.* BL: baseline; W: week; M: mean; SD: standard deviation.

**Table 8:** Statistical significance of the changes in estimated parenting behaviours for intervention and control group.

Cases	Intervention Group				Control Group			
	*df*	Root Mean Square	F	*p*	*df*	Root Mean Square	F	*p*
Supportive/accompanying behaviours	3	13.198	5.264	0.007	3	7.486	2.350	0.114
Residuals	21	2.507			21	3.186		
Punitive/hostile behaviours	3	49.042	27.555	<0.001	3	2.375	1.075	0.389
Residuals	21	1.780			21	2.208		

**Table 9:** Effect size of the programme in family interactions for intervention group.

Supportive/Accompanying Behaviours					Punitive/Hostile Behaviours				
Evaluation Timing	Mean Difference	*t*	Cohen’s d	*p*_holm_	Evaluation Timing	Mean Difference	*t*	Cohen’s d	*p*_holm_
BL.W1.	−1.625	−2.052	−0.726	0.211	BL.W1.	1.875	2.811	0.994	0.021
W2.	−1.875	−2.368	−0.837	0.138	W2.	4.375	6.559	2.319	<0.001
W3.	−3.125	−3.947	−1.395	0.004	W3.	5.500	8.245	2.915	<0.001
W1.W2.	−0.250	−0.316	−0.112	0.755	W1.W2.	2.500	3.748	1.325	0.004
W3.	−1.500	−1.895	−0.670	0.216	W3.	3.625	5.434	1.921	<0.001
W2.W3.	−1.250	−1.579	−0.558	0.259	W2.W3.	1.125	1.687	0.596	0.106

**Table 10:** Effect size of the programme in family interactions for control group.

Supportive/Accompanying Behaviours					Punitive/Hostile Behaviours				
Evaluation Timing	Mean Difference	*t*	Cohen’s d	*p*_holm_	Evaluation Timing	Mean Difference	*t*	Cohen’s d	*p*_holm_
BL.W1.	−0.667	−0.647	−0.264	1.000	BL.W1.	1.167	1.360	0.555	1.000
W2.	−0.333	−0.323	−0.132	1.000	W2.	0.167	0.194	0.079	1.000
W3.	−2.500	−2.426	−0.990	0.170	W3.	1.167	1.360	0.555	1.000
W1.W2.	0.333	0.323	0.132	1.000	W1.W2.	−1.000	−1.166	−0.476	1.000
W3.	−1.833	−1.779	−0.726	0.382	W3.	1.332 × 10^−15^	1.553 × 10^−15^	6.339 × 10^−16^	1.000
W2.W3.	−2.167	−2.102	−0.858	0.264	W2.W3.	1.000	1.166	0.476	1.000

## Data Availability

Not applicable.
